# Association of ZIP code sociodemographic characteristics with radiation oncology services, payments, and technique utilization

**DOI:** 10.3389/fonc.2026.1694910

**Published:** 2026-03-03

**Authors:** Patrick Travis Courtney, Myung-Shin Sim, Michelle A. Eala, Eulanca Y. Liu, Ya-Chen Tina Shih, Michael L. Steinberg, Luca F. Valle, Puja S. Venkat, Michael H. Xiang, Ann C. Raldow

**Affiliations:** 1 Department of Radiation Oncology, University of California, Los Angeles, Los Angeles, CA, United States; 2 Department of Medicine Statistics Core, Division of General Internal Medicine and Health Services Research, University of California, Los Angeles, Los Angeles, CA, United States

**Keywords:** Medicare, radiotherapy payments, radiotherapy services, radiotherapy technique, ZIP code sociodemographics

## Abstract

**Introduction:**

Variations in radiation oncology care by provider and geography have been reported through predominantly analyzed radiotherapy delivery. We evaluated variations in radiation oncologist practices and payments by ZIP code-level sociodemographic data to broaden our understanding of these variations.

**Methods:**

We linked the CMS Medicare Physician and Other Practitioner and National Neighbor Data Archive databases to measure the association between radiation oncologist services, payments, and radiotherapy technique utilization in 2023 and the sociodemographic characteristics of their practice location ZIP code. We included all non-facility radiation oncologists for services and payments and non-facility radiation oncologists who submitted radiotherapy treatment delivery Healthcare Common Procedure Coding System codes for technique utilization. We calculated the percentages of 3D conformal radiotherapy (3DCRT), intensity modulated radiotherapy (IMRT), stereotactic radiosurgery/stereotactic body radiotherapy (SRS/SBRT), proton radiotherapy (PRT), and brachytherapy (BT) use. We used univariable linear regression to measure the association between services, payments, and technique utilization and ZIP code-level sociodemographic variables, and multivariable linear regression to control for ZIP code-level cancer-type proportions.

**Results:**

For services and payments, we identified 2,431 radiation oncologists from 1,126 ZIP codes. In univariable analyses, radiation oncologists in ZIP codes with higher education and income submitted significantly fewer services per beneficiary, as well as fewer unique services per beneficiary for the latter. Increasing ZIP code socioeconomically disadvantaged and Hispanic/Immigrant populations were associated with significantly greater unique services per beneficiary in both univariable and multivariable analyses. Few associations between payments and sociodemographic factors were identified. For technique utilization, we identified 1,400 radiation oncologists from 773 ZIP codes. Radiation oncologists in ZIP codes with a predominantly non-Hispanic White population, higher education, or higher income utilized more SRS/SBRT, PRT, and BT, whereas those in socioeconomically disadvantaged ZIP codes utilized more 3DCRT.

**Discussion:**

This study provides insight into existing inequities in radiation oncology care and suggests that disparities are not limited to care delivery, but exist in radiotherapy technology access, service intensity, and, to a lesser degree, payments, and may be shaped by the sociodemographic context of practice location. These data may serve as a benchmark for measuring the impact of anticipated policy changes on radiotherapy utilization.

## Introduction

It has been well documented that there is considerable variation in healthcare practice and spending across geography in the United States (US), which often cannot be explained by differences in patient or disease characteristics. These differences observed across geographic areas often disproportionately and negatively impact socioeconomically disadvantaged groups and contribute to health inequity and, subsequently, health disparities (1). This topic has been studied broadly in cancer care (2) as well as within radiation oncology. Many studies have reported inequities in the receipt of definitive, high-quality, and/or advanced technique radiotherapy, such as stereotactic body radiotherapy (SBRT) or proton radiotherapy (PRT), by patient sociodemographic factors, particularly race and ethnicity (3–7). Variations in payments and radiotherapy technique utilization by provider characteristics, such as practicing region or sex, have been identified as well (8–10).

However, it is unclear whether these variations extend to radiation oncology services beyond solely technique utilization, and similarly, whether other factors impact radiation oncology payments. Moreover, previous studies have primarily analyzed provider and individual patient-level factors, while additional sociodemographic data—namely, ZIP code-level data—may further our understanding of these variations in radiation oncology services, payments, and technique utilization through evaluation of the broader socioeconomic milieu in which a radiation oncologist practices. Assessing current patterns of physician payment and care in radiation oncology is especially pertinent presently given the anticipated changes to the radiation oncology payment model (11) and recent changes in billing and coding (12). Regarding the former, the Radiation Oncology Case Rate (ROCR) Value-Based Payment Program has been proposed to shift radiation oncology payments from the current fee-for-service model to a bundled, per-patient, or per-episode payment model that will incentivize value over volume and likely impact radiation oncology services and, relatedly, radiotherapy technique utilization as different techniques are required for different regimens such as hypofractionation (11). Regarding radiation oncology billing and coding, the 2026 Centers for Medicare and Medicaid Services (CMS) Hospital Outpatient Prospective Payment System (HOPPS) final rule updated the coding and payments of certain radiation treatment delivery techniques. This will certainly impact radiation oncology payments and, similar to the ROCR model, likely impact services and technique utilization. More broadly, substantial changes to healthcare delivery as a whole in the US are anticipated as well (13).

As such, we sought to evaluate the association of the area-sociodemographic characteristics at the ZIP code level in which a radiation oncologist practices with their services, payments, and radiotherapy technique utilization, hypothesizing that significant variations by ZIP code-level sociodemographic factors exist in these measures.

## Materials and methods

### CMS and NaNDA databases

This is a retrospective, cross-sectional study using publicly available data. For radiation oncologist payment and practice data, we used the CMS Medicare Physician and Other Practitioner (MPOP) “By Provider and Service” dataset, which contains information on the services provided to Original Medicare (fee-for-service) Part B beneficiaries and the associated payments received by providers, aggregated by provider and service (14). Medicare is a federal health insurance program for U.S. citizens aged 65 years or older (90% of enrollees) (15), or those with disabilities or certain health conditions (16). Medicare insurance consists of four parts (A, B, C, and D) related to the general type of service covered, and Part B covers outpatient services. As of 2026, approximately 34 million U.S. citizens (10% of the U.S. population) are enrolled in “Original” or “Traditional” Medicare, which includes Part B. In the MPOP dataset, providers are identified by their National Provider Identifier (NPI) number registered in the National Plan and Provider Enumerator System (NPPES), and their specialty is derived from the provider specialty code reported on the service claim, or in the case of multiple reported specialty codes, the specialty associated with the largest number of services. Providers are also categorized by their place of service, defined as either facility or non-facility. Facility-based settings are hospital-based whereas non-facility-based settings include a variety of practice types such as independent outpatient clinics, federally qualified health centers, and rural health clinics (17). Additionally, dataset includes the provider’s ZIP code as reported in the NPPES. In this study, we utilized service and payment data from 1 January 2023 to 31 December 2023. Services and payments provided in facility settings were excluded as these payments only include physician’s professional fees in the MPOP dataset, whereas services and payments provided in non-facility settings represent complete payment for the service (9, 14). For Medicare beneficiary privacy, records that are aggregated from 10 or fewer beneficiaries are excluded from the MPOP dataset.

**Figure 1:**
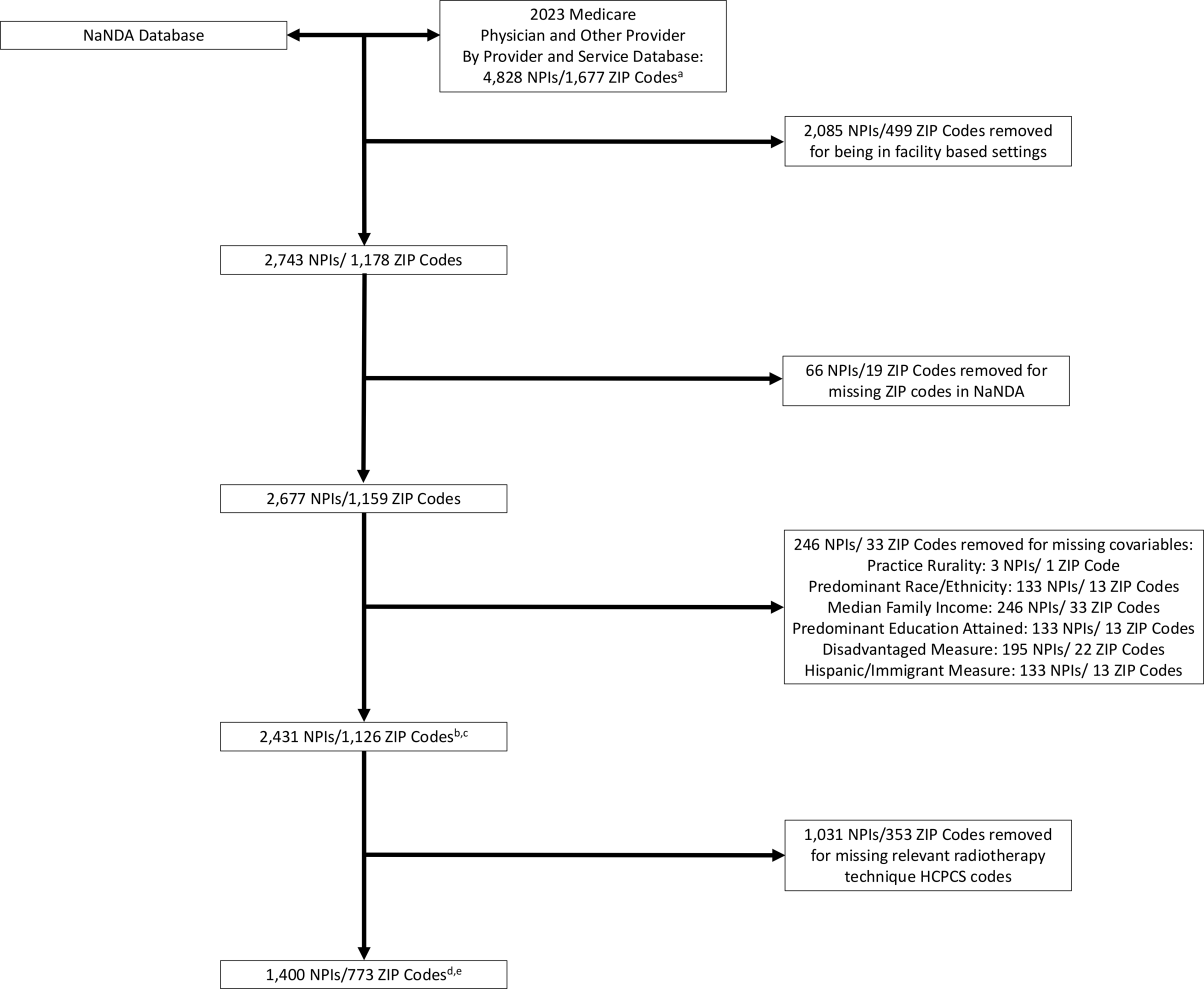
Flowchart of services, payments, and radiotherapy technique analyses cohorts. ^a^With provider specialty listed as “Radiation Oncology”. ^b^For services and payments analyses. ^c^For sensitivity analyses of services and payments incorporating ZIP code cancer proportions from the SEER database, 1,387 NPIs/645 ZIP codes were removed as they were not available in SEER, yielding 1,044 NPIs/481 ZIP codes for these analyses. ^d^For radiotherapy technique utilization analyses. ^e^For sensitivity analyses of radiotherapy technique utilization incorporating ZIP code cancer proportions from the SEER database, 821 NPIs/454 ZIP codes were removed as they were not available in SEER, yielding 579 NPIs/316 ZIP codes for these analyses.

For ZIP code-level sociodemographic data, we utilized the National Neighbor Data Archive (NaNDA) Socioeconomic Status and Demographic Characteristics dataset (18). This dataset, which was created by the University of Michigan Institute for Social Research, contains tabulation of US Census Tract and ZIP code area socioeconomic and demographic measures from 1990 to 2022 from the American Community Survey. The CMS MPOP dataset was linked to the NaNDA dataset by provider ZIP code to associate CMS provider payment and service data with the ZIP code-level sociodemographic characteristics associated with their practice location. Radiation oncologists with ZIP codes that were missing in the NaNDA dataset were excluded from the analysis (**Figure 1**).

### Objectives

The main objectives of this study were to measure associations between radiation oncologist ZIP code sociodemographic characteristics with Original Medicare Part B (1) services submitted, (2) payments received, and (3) radiotherapy technique utilization. For services, we calculated each provider’s total services, representative of productivity (8), and unique services, representative of breadth of practice (8). To account for differences in radiation oncologist patient population size, services and payments were calculated per beneficiary. For radiotherapy technique utilization, we included only radiation oncologists who submitted radiotherapy treatment delivery Healthcare Common Procedure Coding System (HCPCS) codes (19) (**Figure 1** and **Supplementary Table 1**). Five separate radiotherapy techniques were analyzed: (1) 3D conformal radiotherapy (3DCRT), (2) intensity modulated radiotherapy (IMRT), (3) stereotactic radiosurgery (SRS)/SBRT, (4) PRT, and (5) brachytherapy (BT).

For each radiation oncologist, utilization of a particular radiotherapy technique was calculated as the percentage of beneficiaries receiving an individual radiotherapy technique divided by the number of beneficiaries receiving any of the five radiotherapy techniques. For example, if 10 beneficiaries received 3DCRT and 10 received IMRT for one radiation oncologist, the 3DCRT percentage would be 50%, the IMRT percentage would be 50%, and the SRS/SBRT, PRT, and BT percentages would be 0%.

### Sociodemographic variables

The sociodemographic variables of interest were ZIP code predominant race/ethnicity (Hispanic, non-Hispanic Black, or non-Hispanic White), ZIP code predominant education attained (less than high school diploma/high school diploma/some college or Bachelor’s degree or higher), and median family annual income. ZIP code predominant race/ethnicity and education attained was derived from the aforementioned categories with the highest percentage in the ZIP code. Median family income was grouped into quartiles.

The NaNDA dataset additionally contains a theoretically derived composite measure of ZIP code “disadvantage”, calculated as the mean of the ZIP code proportions of Black people, female-headed families with kids, households with public assistance income or food stamps, people with income in the past 12 months below the poverty level, and age 16+ civilian labor force unemployed. The NaNDA data derived another composite measure of “Hispanic/Immigrant”, calculated as the mean of the ZIP code proportions of Hispanic people, people who are foreign born, and people who speak limited English. Both composite measures were included as variables of interest.

Finally, the CMS MPOP dataset contains provider practice rurality, which was also included as a variable of interest in this study. Practice rurality was defined as either rural or metropolitan according to the U.S. Department of Agriculture Rural–Urban Commuting Areas Codes (20). Radiation oncologists with ZIP codes that were missing any of the sociodemographic variables of interest were excluded from the analysis (**Figure 1**).

### Statistical analysis

We used univariable linear regression to measure the association between services, payments, and radiotherapy technique percentages and our area-based sociodemographic variables of interest. For the radiotherapy technique analyses, each radiotherapy technique was analyzed independently. Multivariable linear regression incorporating multiple sociodemographic variables was not performed because (1) of significant associations between sociodemographic variables on chi-square testing (**Supplementary Data 1**), consistent with previous reports (21–27), and (2) the disadvantage and Hispanic/Immigrant composite measures were able to account for the association of a combination of sociodemographic measures.

To address potential confounding from differences in individual ZIP code cancer-type incidences, we performed sensitivity analyses utilizing multivariable linear regression that included ZIP code-level proportions of the 13 most incident cancers in the US in 2025 (28) (breast, colorectal, kidney and renal pelvis, leukemia, lung and bronchus, lymphoma, melanoma of the skin, oral cavity and pharynx, pancreas, prostate, thyroid, urinary bladder, and uterine corpus), which were obtained from the most recent Surveillance, Epidemiology and End Results (SEER) Program, SEER Research Plus Data (29). These data were linked through a FIPS-ZIP code crosswalk (30). Proportions of ZIP code cancer types were calculated as the annual incidence of that cancer type divided by the ZIP code total population. Radiation oncologists with ZIP codes that were not included in SEER were excluded from this sensitivity analysis (**Figure 1**).

Statistical analyses were performed with SAS V9.4 (SAS Institute, Cary, NC) and assumed a two-sided alpha of 0.05.

## Results

### Baseline cohorts

We identified 2,431 radiation oncologists from 1,126 unique ZIP codes for the services and payments analyses, and 1,400 radiation oncologists from 773 unique ZIP codes for the radiotherapy technique utilization analyses. **Table 1** shows the baseline characteristics of the two datasets used in the services, payments, and technique utilization analyses. In both datasets, the majority of radiation oncologists practiced in ZIP codes that were metropolitan, had a predominantly non-Hispanic White population, had a predominant population who attained less than high school diploma/high school diploma/some college of education, and had a mean median family annual income slightly greater than $100,000. The mean proportions of the disadvantage and Hispanic/Immigrant measures were approximately 18% and 12%, respectively.

**Table 1:** Cohort characteristics.

Characteristics	For service and payment analyses (2,431 NPIs/1,126 ZIP codes)	For radiotherapy technique analysis (1,400 NPIs/773 ZIP codes)
Mean population size, *n* (IQR)	31,185 (19,071–40,598)	31,877 (19,744–40,923)
Practice rurality		
Metropolitan, *n* (%)	2,279 (93.7)	1,303 (93.7)
Rural, *n* (%)	152 (6.3)	97 (6.9)
Predominant race/ethnicity		
Hispanic, *n* (%)	272 (11.2)	159 (11.4)
Non-Hispanic Black, *n* (%)	188 (7.7)	108 (7.7)
Non-Hispanic White, *n* (%)	1,971 (81.1)	1,133 (80.9)
Predominant education attained		
Less than high school diploma, high school diploma, and/or some college, *n* (%)	1,386 (57.0)	852 (60.9)
Bachelor’s degree or higher, *n* (%)	1,045 (43.0)	548 (39.1)
Mean ZIP code median family annual income, $ (IQR)	107,794 (72,159–129,343)	104,820 (73,817–126,616)
Mean ZIP code “disadvantage” measure percentage (IQR)	18.4 (10.7–24.3)	17.9 (11.2–23.3)
Mean ZIP code “Hispanic/Immigrant” measure percentage (IQR)	11.8 (4.8–15.0)	11.6 (4.8–15.4)
Mean number of beneficiaries (IQR)	652 (124–963)	108 (35–156)^a^
Mean total number of services per beneficiary (IQR)	3.7 (2.1–4.2)	10.5 (4.1–16.1)^a^
Mean unique services submitted per beneficiary (IQR)	0.03 (0.02–0.05)	--
Mean payment per beneficiary, $ (IQR)	470 (167–678)	--
Mean 3DCRT percentage (IQR)	--	23.1 (0–40.8)
Mean IMRT percentage (IQR)	--	53.9 (38.1–76.5)
Mean SRS/SBRT percentage (IQR)	--	4.9 (0–8.9)
Mean PRT percentage (IQR)	--	5.7 (0–0)^b^
Mean BT percentage (IQR)	--	12.4 (0–0)^c^

3DCRT, 3D conformal radiotherapy; BT, brachytherapy; HCPCS, healthcare common procedure coding system; IMRT, intensity modulated radiotherapy; IQR, interquartile range; NPI, national provider identifier; PRT, proton radiotherapy; SBRT, stereotactic body radiotherapy, SEER, Surveillance, Epidemiology and End Results; SRS, stereotactic radiosurgery.

a Who received the radiotherapy treatment delivery HCPCS codes of interest.

b A total of 94 NPIs/46 ZIP codes had PRT percentage >0.

c A total of 250 NPIs/194 ZIP codes had PRT percentage >0.

For the services submitted analysis, the mean number of total and unique services submitted per beneficiary per radiation oncologist were 3.7 and 0.03, respectively. For the payment analysis, the mean total payment per beneficiary per radiation oncologist was $470. For the radiotherapy technique analysis, the most common technique was IMRT (53.9%), followed by 3DCRT (23.1%), BT (12.4%), PRT (5.7%), and SRS/SBRT (4.9%).

### Services/productivity

Compared with radiation oncologists in ZIP codes with a predominantly Hispanic population, radiation oncologists in ZIP codes with predominantly non-Hispanic Black or White populations submitted significantly fewer unique services per beneficiary (**Table 2**). Radiation oncologists in ZIP codes with a predominant population who attained at least a Bachelor’s degree of education submitted significantly fewer total services per beneficiary, although their unique services per beneficiary were significantly greater. Increasing ZIP code median family income quartile was associated with significantly fewer unique services per beneficiary and, at the highest quartile, significantly fewer total services submitted. Conversely, increasing ZIP code disadvantage and Hispanic/Immigrant measures were associated with significantly greater unique services per beneficiary.

**Table 2:** Univariable linear regression analyses of association between radiation oncologist services and payments and ZIP code sociodemographic factors.

Variable	Total services per beneficiary coefficient (95% CL)	*P*-value	Unique services per beneficiary coefficient (95% CL)	*P*-value	Payment per beneficiary coefficient (95% CL)	*P*-value
Practice rurality						
Metropolitan (reference)	--		--		--	
Rural	0.1 (−0.5–0.8)	0.67	0.0 (−0.003–0.003)	0.94	45.3 (38.9–129.7)	0.29
Predominant race/ethnicity						
Hispanic (reference)	--		--		--	
Non-Hispanic Black	−0.7 (−1.4–0.02)	0.058	**−0.004 (−0.01**–**0.001)**	**0.021**	7.9 (−87.6–103.4)	0.87
Non-Hispanic White	−0.4 (−0.9–0.1)	0.10	**−0.005 (−0.008 to −0.003)**	**<0.0001**	−3.7 (−68.8–61.5)	0.91
Predominant education attained						
Less than high school diploma, high school diploma, and/or some college (reference)	--		--		--	
Bachelor’s degree or higher	**−0.4** (−**0.7 to −0.1)**	**0.009**	**0.002 (0.0001**–**0.003)**	**0.041**	**−73.6** (−**114.8 to −32.5)**	**0.0005**
Median annual family income, $ (range)						
Quartile 1 (2,499–73,555) (reference)	--		--		--	
Quartile 2 (73,602–94,487)	−0.02 (−0.5–0.4)	0.92	**−0.004 (−0.006 to −0.002)**	**0.0005**	24.3 (−34.0–82.5)	0.41
Quartile 3 (94,592–125,703)	0.1 (−0.3–0.5)	0.68	**−0.003 (−0.005 to −0.001)**	**0.020**	−19.8 (−77.2–37.6)	0.50
Quartile 4 (125,861–250,001)	**−0.6 (−1.0 to −0.1)**	**0.009**	**−0.002 (−0.004 to −0.001)**	**0.038**	−51.9 (−107.5–4.3)	0.071
**Increasing ZIP code “disadvantage” measure**	−0.3 (−1.9–1.3)	0.68	**0.02 (0.01**–**0.03)**	**<0.0001**	−85.3 (−297.0–126)	0.43
**Increasing ZIP code “Hispanic/Immigrant” measure**	0.8 (−0.8–2.5)	0.31	**0.02 (0.01**–**0.03)**	**<0.0001**	30.0 (−187.2–247.3)	0.79

CL, confidence limits.

Bold values are statistically significant with a p-value <0.05.

In the sensitivity analyses controlling for SEER ZIP code cancer proportions (**Table 3**), ZIP codes with a predominantly non-Hispanic White population remained significantly associated with fewer unique services per beneficiary compared with ZIP codes with a predominantly Hispanic population, as did increasing ZIP code disadvantage and Hispanic/Immigrant measures.

**Table 3:** Multivariable linear regression analyses of association between radiation oncologist services and payments and ZIP code sociodemographic factors, controlling for ZIP code cancer proportions from the SEER database.

Variable	Total services per beneficiary coefficient (95% CL)	*P*-value	Unique services per beneficiary coefficient (95% CL)	*P*-value	Payment per beneficiary coefficient (95% CL)	*P*-value
Practice rurality						
Metropolitan (reference)	--		--		--	
Rural	−0.2 (−1.0–0.6)	0.62	0.0 (−0.01–0.02)	0.34	31.5 (−69.8–132.9)	0.54
Predominant race/ethnicity						
Hispanic (reference)	--		--		--	
Non-Hispanic Black	−0.7 (−1.5–0.2)	0.12	0.0 (−0.01–0.03)	0.54	−**113.3 (−221.9 to −4.6)**	**0.041**
Non-Hispanic White	−0.1 (−0.6–0.4)	0.76	**−0.004 (−0.007 to −0.001)**	**0.009**	−14.7 (−80.8–51.3)	0.66
Predominant education attained						
Less than high school diploma, high school diploma, and/or some college (reference)	--		--		--	
Bachelor’s degree or higher	−0.1 (−0.5–0.3)	0.57	0.0 (−0.01–0.03)	0.73	−45.2 (−95.6–5.2)	0.08
Median annual family income, $ (range)						
Quartile 1 (2,499–73,555) (reference)	--		--		--	
Quartile 2 (73,602–94,487)	0.0 (−0.5–0.6)	0.88	0.0 (−0.01–0.01)	0.35	48.6 (−23.4–120.7)	0.19
Quartile 3 (94,592–125,703)	0.4 (−0.2–0.9)	0.18	0.0 (−0.01–0.01)	0.17	40.2 (−32.1–112.4)	0.28
Quartile 4 (125,861–250,001)	−0.2 (−0.7–0.3)	0.42	0.0 (−0.01–0.01)	0.36	1.6 (−65.3–68.5)	0.96
**Increasing ZIP code “disadvantage” measure**	−0.3 (−2.2–1.7)	0.79	**0.01 (0.001**–**0.02)**	**0.03**	−32.8 (−286.5–220.9)	0.80
**Increasing ZIP code “Hispanic/Immigrant” measure**	0.7 (−1.5–3.0)	0.52	**0.03 (0.01**–**0.04)**	**<0.0001**	12.7 (−284.7–310.2)	0.93

CL, confidence limits.

Bold values are statistically significant with a p-value <0.05.

### Payments

Radiation oncologists in ZIP codes with a predominant population who attained at least a Bachelor’s degree of education received significantly fewer payments per beneficiary (**Table 2**). In the sensitivity analyses controlling for SEER ZIP code cancer proportions (**Table 3**), compared with radiation oncologists in ZIP codes with a predominantly Hispanic population, radiation oncologists in ZIP codes with a predominantly non-Hispanic Black population received significantly fewer payments per beneficiary. No other sociodemographic variable was associated with payments per beneficiary in the payment analyses.

### Radiotherapy technique

Radiation oncologists in rural practices utilized significantly greater percentages of 3DCRT and significantly lower percentages of SRS/SBRT and PRT (**Table 4**). Compared with radiation oncologists in ZIP codes with a predominantly Hispanic population, radiation oncologists practicing in ZIP codes with a predominantly non-Hispanic Black population utilized significantly lower percentages of IMRT and significantly greater percentages of PRT, whereas radiation oncologists practicing in predominantly non-Hispanic White ZIP codes utilized significantly greater percentages of SRS/SBRT. Radiation oncologists practicing in ZIP codes with a predominant population who attained at least a Bachelor’s degree of education utilized significantly lower percentages of 3DCRT and IMRT and significantly greater percentages of PRT and BT. For ZIP code income, compared with the lowest quartile, the highest quartile was significantly associated with lower 3DCRT and greater BT utilization. Increasing ZIP code disadvantage measure was associated with significantly lower IMRT utilization and significantly greater PRT utilization, while increasing ZIP code Hispanic/Immigrant measure was associated with significantly lower SRS/SBRT utilization.

**Table 4:** Univariable linear regression analyses of association between radiotherapy technique utilization ZIP code sociodemographic factors.

Variable	3DCRT coefficient (95% CL)	*P*-value	IMRT coefficient (95% CL)	*P*-value	SRS/SBRT coefficient (95% CL)	*P*-value	PRT coefficient (95% CL)	*P*-value	BT coefficient (95% CL)	*P*-value
Practice rurality										
Metropolitan (reference)	--		--		--		--		--	
Rural	**5.1** **(0.3**–**9.8)**	**0.038**	6.7 (−0.08–13.4)	0.053	**−2.8** **(−4.8 to −0.8)**	**0.006**	**−5.0** **(−9.5 to −0.5)**	**0.029**	**−**3.9 (−10.4–2.6)	0.24
Predominant race/ethnicity										
Hispanic (reference)	--		--		--		**--**		--	
Non-Hispanic Black	**−**3.2 (−8.9–2.4)	0.26	**−8.8** **(−16.8 to −0.8)**	**0.031**	1.8 (−0.5–4.2)	0.13	**7.5** **(2.2**–**12.8)**	**0.006**	2.7 (−5.0–10.5)	0.49
Non-Hispanic White	0.6 (−3.2–4.4)	0.76	**−**3.4 (−8.9–2.0)	0.22	**1.8** **(0.2**–**3.4)**	**0.030**	−0.8 (−4.4–2.9)	0.66	1.8 (−3.4–7.1)	0.50
Predominant education attained										
Less than high school diploma, high school diploma, and/or some college (reference)	--		--		--		--		--	
Bachelor’s degree or higher	**−8.8** **(−11.2 to −6.3)**	**<0.0001**	**−5.1** **(−8.6 to −1.6)**	**0.005**	−0.2 (−1.2–0.9)	0.75	**4.4** **(2.1**–**6.8)**	**0.0002**	**9.6** **(6.2**–**12.9)**	**<0.0001**
Median annual family income, $ (range)										
Quartile 1 (32,340–73,817) (reference)	--		--		--		--		--	
Quartile 2 (73,909–93,694)	−0.1 (−3.5–3.3)	0.96	**7.4** **(2.6**–**12.2)**	**0.003**	0.4 (−1.0–1.9)	0.54	**−3.9** **(−7.1 to −0.6)**	**0.019**	03.9 (−8.6–0.8)	0.10
Quartile 3 (93,703–126,607)	**−**1.4 (−4.9–2.0)	0.41	**5.8** **(1.0**–**10.7)**	**0.018**	0.8 (−1.8–1.1)	0.29	**−3.3** **(−6.6 to −0.1)**	**0.041**	**−**1.8 (−6.5–2.9)	0.44
Quartile 4 (126,625–250,001)	**−5.9** **(−9.4 to −2.5)**	**0.0007**	0.4 (−4.4–5.3)	0.86	−0.3 (−1.8–1.1)	0.66	1.2 (−2.1–4.4)	0.48	**4.7** **(0.0**–**9.3)**	**0.0496**
**Increasing ZIP code disadvantage measure**	7.1 (−6.3–20.4)	0.30	**−22.2** **(−41.1 to −3.3)**	**0.02**	**−**2.2 (−7.8–3.4)	0.45	**20.8** **(8.3**–**33.4)**	**0.001**	**−**3.5 (−21.8–14.7)	0.71
**Increasing ZIP code Hispanic/Immigrant measure**	−0.9 (−14.5–23.6)	0.89	9.9 (−9.2–29.0)	0.31	**−6.2** **(−11.8 to −0.5)**	**0.032**	**−**8.8 (−21.5–4.0)	0.18	6.0 (−12.5–24.4)	0.53

3DCRT, 3D conformal radiotherapy; BT, brachytherapy; CL, confidence limits; IMRT, intensity modulated radiotherapy; PRT, proton radiotherapy; SBRT, stereotactic body radiotherapy, SEER, Surveillance, Epidemiology and End Results; SRS, stereotactic radiosurgery.

Bold values are statistically significant with a p-value <0.05.

In the sensitivity analyses controlling for SEER ZIP code cancer proportions (**Table 5**), rural practice remained associated with significantly greater 3DCRT and lower SRS/SBRT utilization. While predominantly non-Hispanic White populations remained associated with greater percentages of SRS/SBRT, the associations between a predominantly non-Hispanic Black population and IMRT and PRT utilization were no longer present. Increasing ZIP code disadvantage measure became significantly associated with greater percentages of 3DCRT, and the associations between IMRT and PRT were no longer significant.

**Table 5:** Multivariable linear regression analyses of association between radiotherapy technique utilization and ZIP code sociodemographic factors, controlling for ZIP code cancer proportions from the SEER database.

Variable	3DCRT coefficient (95% CL)	*P*-value	IMRT coefficient (95% CL)	*P*-value	SRS/SBRT coefficient (95% CL)	*P*-value	PRT coefficient (95% CL)	*P*-value	BT coefficient (95% CL)	*P*-value
Practice rurality										
Metropolitan (reference)	--		--		--		--		--	
Rural	**12.4** **(5.4**–**19.4)**	**0.0006**	**−**1.7 (−12.0–8.5)	0.73	**−3.0** **(−5.8 to −0.2)**	**0.033**	**−**1.2 (−7.7–5.3)	0.72	**−**6.4 (−16.0–3.2)	0.19
Predominant race/ethnicity										
Hispanic (reference)	--		--		--		--		--	
Non-Hispanic Black	8.3 (−1.0–17.3)	0.07	−0.2 (−13.2–12.8)	0.98	−0.3 (−3.8–3.3)	0.89	**−**6.1 (−14.3–2.2)	0.15	**−**1.8 (−14.0–10.4)	0.78
Non-Hispanic White	1.8 (−3.0–6.7)	0.45	1.7 (−5.4–8.7)	0.64	**2.4** **(0.5**–**4.3)**	**0.014**	**−**4.4 (−8.9–0.03)	0.052	**−**1.5 (−8.1–5.1)	0.66
Predominant education attained										
Less than high school diploma, high school diploma, and/or some college (reference)	--		--		--		--		--	
Bachelor’s degree or higher	**−8.1** **(−11.7 to −4.4)**	**<0.0001**	**−5.4** **(−10.7 to −0.03)**	**0.048**	0.6 (−0.9–2.0)	0.44	**5.0** **(1.6**–**8.4)**	**0.004**	**7.9** **(2.9**–**12.8)**	**0.002**
Median annual family income, $ (range)										
Quartile 1 (32,340–73,817) (reference)	--		--		--		--		--	
Quartile 2 (73,909–93,694)	1.4 (−3.5–6.4)	0.57	3.5 (−3.7–10.7)	0.34	0.1 (−1.9–2.1)	0.90	**−**3.2 (−7.8–1.4)	0.17	**−**1.8 (−8.6–4.9)	0.59
Quartile 3 (93,703–126,607)	**−**2.6 (−7.7–2.4)	0.31	4.4 (−2.9–11.7)	0.23	0.5 (−1.5–2.5)	0.64	**−**3.3 (−7.9–1.4)	0.17	1.0 (−5.8–7.8)	0.78
Quartile 4 (126,625–250,001)	**−8.2** **(−13.3 to −3.1)**	**0.002**	**−**7.2 (−14.6–0.3)	0.058	0.2 (−1.8–2.3)	0.83	3.6 (−1.1–8.4)	0.13	**11.5** **(4.6**–**18.4)**	**0.001**
**Increasing ZIP code disadvantage measure**	**20.7** **(1.1**–**40.4)**	**0.038**	**−**15.5 (−43.9–12.9)	0.28	0.5 (−7.2–8.3)	0.89	13.8 (−4.2–31.8)	0.13	**−**19.6 (−46.1–7.0)	0.15
**Increasing ZIP code Hispanic/Immigrant measure**	**−26.9** **(−48.9 to −4.9)**	**0.017**	**−**4.0 (−35.9–27.9)	0.80	**−15.8** **(−24.5 to −7.2)**	**0.0004**	15.4 (−4.8–35.7)	0.13	**31.2** **(1.4**–**61.0)**	**0.040**

3DCRT, 3D conformal radiotherapy; BT, brachytherapy; CL, confidence limits; IMRT, intensity modulated radiotherapy; PRT, proton radiotherapy; SBRT, stereotactic body radiotherapy, SEER, Surveillance, Epidemiology and End Results; SRS, stereotactic radiosurgery.

Bold values are statistically significant with a p-value <0.05.

## Discussion

In this analysis, we comprehensively evaluated radiation oncologist services, payments, and technique utilization on the national level to gain insight into the existence of variation in these measures according to ZIP code sociodemographic metrics. To our knowledge, the current study is the first to utilize this novel combination of datasets to analyze variations in radiation oncology practice patterns. These data suggest that systemic disparities not just in care delivery but also in technology access, service intensity, and, to a lesser degree, radiation oncologist payments may be shaped by the sociodemographic context of practice location. Importantly, these data serve as an important benchmark for future studies of radiation oncology services in light of the recent and potential upcoming changes to radiation oncology billing and payment (11, 12), in addition to general access to healthcare in the US (13).

In the services analyses, certain findings from the current study appear counterintuitive to existing data on the association between sociodemographic factors and radiation oncology care, namely, the negative association between more “advantageous” sociodemographic factors such as higher ZIP code education or income and radiation oncologist productivity and breadth of service. Simultaneously, increasing ZIP code disadvantage and Hispanic/Immigrant measures were associated with increased breadth of service, which is seemingly in contrast to previous studies demonstrating that such populations typically have decreased healthcare access and utilization (31–37). One consideration is that socioeconomically advantageous factors are associated with earlier stage at diagnosis (38–40), which may require shorter, less intensive radiotherapy regimens and incur fewer long-term disease-related costs related to toxicity or recurrence, overall resulting in lower services and payments per beneficiary. However, the reverse may also be true, whereby patients presenting with more advanced-stage disease, particularly metastatic disease, undergo shorter courses of palliative radiotherapy compared with relatively longer definitive radiotherapy courses for patients with non-metastatic disease. Another possibility is that radiation oncologists practicing in more socioeconomically advantaged ZIP codes may be affiliated with academic centers or large integrated healthcare systems that emphasize value, evidence-based care, shorter fractionation regimens, and cost containment, whereas other non-facility-based clinics working to maintain their independence may be more subjected to financial pressures and subsequently utilize longer fractionation courses (41), though the datasets used in this study do not provide insight into the financial considerations of the radiation oncologists or their clinics. Finally, more socioeconomically advantaged populations may have more resources to travel outside their ZIP code of residence to tertiary and/or academic medical centers, which are facility-based and thus not included in the current study, which may artificially lower the total and unique services provided by radiation oncologists in more advantaged ZIP codes. Notably, the associations between ZIP code education and income and services did not persist when controlling for ZIP code cancer proportions, suggesting that disparities in cancer incidences by sociodemographic characteristics (40) contribute to differences in radiation oncology service utilization, although this sensitivity analysis excluded numerous providers due to unavailable ZIP codes in SEER. Ultimately, this finding may be at least in part artifactual given the limitations of the datasets used in this study, described below.

When analyzing payments, few associations were identified, most notably decreased payments per beneficiary in ZIP codes with a predominantly non-Hispanic Black versus Hispanic population. Additionally, as with the services analyses, higher ZIP code education was associated with fewer payments per beneficiary on univariable analysis, which again may be due to multiple factors noted in the preceding paragraph that reduce service intensity and, in turn, lower payments per beneficiary without necessarily indicating under-treatment. An optimistic interpretation of these findings is that previously identified sociodemographic inequities in cancer care access and utilization (40), which likely translate to inequities in provider payments, have improved in recent years, though, to our knowledge, there are limited data (42) describing the relationship between radiation oncologist payments and the sociodemographic makeup of their practicing ZIP code with which to contextualize our results. Moreover, the current study does not include commercial insurance data, including Medicare Advantage, and thus is not fully inclusive of all payments received. Disparities in commercial insurance coverage (43, 44) and policies (45, 46) may result in differences in provider payments by ZIP code-level sociodemographic factors and warrant further study.

Regarding radiotherapy technique utilization, we found that the use of more advanced, resource-intensive techniques, such as SRS/SBRT, PRT, and BT, was positively associated with measures of socioeconomic advantage, such as a predominantly non-Hispanic White population, increasing income, or higher education attained. On the other hand, 3DCRT, a less advanced technique, was more frequently utilized in ZIP codes with increasing disadvantage measure and rural practices, as was SRS/SBRT with the latter. While similar findings have been reported in other studies of inequities in radiation oncology (3–10), these data provide additional context on these relationships and, given their derivation from recently generated datasets, demonstrate that many inequities in the radiotherapy technique persist despite increased awareness. Contrary to prior data, we found that ZIP codes with a predominantly non-Hispanic Black population or with an increasing disadvantage measure had significantly greater PRT utilization in the univariable analyses; however, these associations were no longer present when incorporating ZIP code cancer proportions. This may be at least in part explained by the increased incidence of prostate cancer in Black patients (47), which is one of the cancer types most frequently treated with PRT (48). It should be noted, however, that utilization of certain advanced techniques does not necessarily represent improved access or more effective care. For example, while increased PRT use may reflect better access to this technology, it may also reflect unnecessary overutilization of a resource-intensive radiotherapy modality when less advanced techniques such as 3DCRT or IMRT suffice.

A key consideration when evaluating this study is the understanding that provider ZIP codes do not necessarily correlate with patient ZIP codes, and as such, ZIP code-level practices may not fully reflect the sociodemographic milieu. While prior studies have found that approximately 78% of the U.S. population live within 12.5 miles of a radiotherapy facility (49) and that the median patient travel distance to radiotherapy is 20 miles (50), substantial ZIP code-level sociodemographic variation can exist across these distances. This may skew the data presented in this study as a result of patients being treated in ZIP codes that do not resemble the ZIP codes in which they reside, which is ultimately the question this study seeks to answer. For example, socioeconomically advantaged ZIP codes may have referral centers with a particular focus on certain radiotherapy techniques (SRS/SBRT, PRT, and BT) in which patients are being treated from afar, or vice versa, a more socioeconomically advantaged population with the resources to travel to faraway radiotherapy centers. Despite this distinction between provider and patient ZIP codes, there likely exists some overlap in the sociodemographic breakdown between radiation oncologist and patient ZIP codes given that the two are often in geographic proximity.

This study has multiple limitations. This study does not include Medicaid or commercial insurance data, including from Medicare Advantage plans in which enrollment has steadily increased in recent years (51). Furthermore, almost all Medicare beneficiaries are 65 or older, and thus, the associations found in the current study may differ in other settings, such as the commercially insured or younger populations. Nonetheless, Medicare data remain highly relevant in oncology, as Medicare covers a substantial proportion of U.S. patients with cancer. Additionally, prior work suggests that Medicare data may, if anything, underestimate variation in practice patterns compared with commercial insurance populations (52). Similarly, restricting the analysis to non-facility-based services limits the generalizability of our findings. **Figure 1** shows that approximately 50% of radiation oncologists in the 2023 MPOP dataset provided care in the non-facility setting, a proportion consistent with prior descriptions of the U.S. radiation oncology workforce (9, 10, 53). Although this approach excludes a substantial subset of practicing radiation oncologists, we believe that the included cohort nonetheless represents a meaningful segment of the specialty from which practice patterns can be assessed. This is particularly relevant in a relatively small field such as radiation oncology, where national trends can often be observed within defined subsets of practice. Moreover, prior studies using similar CMS-based methodologies have demonstrated comparable patterns and associations across facility and non-facility settings (8, 10). Relatedly, approximately 9% of non-facility-based radiation oncologists (5% of the entire MPOP dataset) were excluded because of missing ZIP code-level data. Regarding the SEER analyses, these were included as sensitivity analyses to further contextualize and support our primary findings. Although SEER has well-recognized limitations related to its geographic coverage, it remains a widely used and authoritative dataset in health services research and cancer epidemiology. We believe that meaningful insights can still be drawn from these sensitivity analyses despite the exclusion of radiation oncologists practicing in ZIP codes not captured by SEER. These analyses were intended to supplement, rather than replace, our primary analyses and do not constitute the entirety of the data underlying our conclusions. Other relevant data such as treatment indication and regimen are also unavailable in the datasets used in the current study, and it is not possible to determine whether treatment decisions were made according to guideline recommendations, technology or resource availability, and/or patient and provider preferences. There are likely additional unmeasured and potentially confounding sociodemographic variables that are associated with radiation oncology practice patterns that were unavailable in the NaNDA dataset. Ultimately, the decision to utilize certain radiotherapy services or techniques is an amalgam of components ranging from the patient to the healthcare system level in which sociodemographic factors likely play a crucial role. Finally, inclusion of multiple comparisons may risk generating spurious, statistically significant findings.

In conclusion, this study demonstrated that radiation oncologist services, radiotherapy technique utilization, and, to a lesser degree, payments vary with certain ZIP code-level sociodemographic characteristics, furthering our understanding of the relationship between these factors on radiation delivery. These data not only identify possible existing inequities in radiation oncology care that need to be addressed but also may allow future researchers to assess the impact of policy changes on radiotherapy utilization.

## Author’s note

A previous version of this project was presented as a poster and published as an online-only abstract as below:

P.T. Courtney, L. Valle, A. Raldow. Association of Provider Zip Code Sociodemographic Characteristics with Radiation Therapy Modality Use in the Medicare Population. American Society for Radiation Oncology (ASTRO) 2023 Annual Meeting. International Journal of Radiation Oncology*Biology*Physics, Volume 117, Issue 2, Supplement, 2023, Page e13, ISSN 0360-3016. https://doi.org/10.1016/j.ijrobp.2023.06.675. October 1, 2023.

## Data Availability

The raw data supporting the conclusions of this article will be made available by the authors, without undue reservation.
