# Supplementary material for: Association of ZIP code sociodemographic characteristics with radiation oncology services, payments, and technique utilization
Source: Front Oncol. 2026 Mar 3;16:1694910. doi: 10.3389/fonc.2026.1694910 (PMC12991990; doi:10.3389/fonc.2026.1694910)
Supplement: Supplementary file 2 [file Table1.docx]

**Supplementary Table 1**: Healthcare Common Procedure Code System (HCPCS) codes used to identify radiotherapy delivery technique.

| **Radiotherapy Technique** | **HCPCS Codes** |
| --- | --- |
| 3D Conformal Radiotherapy (3DCRT) | 77401-77416, G6003-G6014 |
| Intensity Modulated Radiotherapy (IMRT) | 77385, 77386, G6015, G6016 |
| Stereotactic Body Radiotherapy (SBRT)/Stereotactic Radiosurgery (SRS) | 77371-77373 |
| Proton Radiotherapy (PRT) | 77520-77525 |
| Brachytherapy (BT) | 77750, 77761-77763, 77767, 77768, 77770, 77771, 77772, 77778, 77789, 77790, 0394T, 0395T |
